# Student Perception and Preferences With Social Media for Enhanced Learning in Health Sciences Following Post-COVID-19 Era: A Cross-Sectional Study

**DOI:** 10.7759/cureus.47390

**Published:** 2023-10-20

**Authors:** Osama Khattak, Kiran K Ganji, Anshoo Agarwal, Azhar Iqbal, Mahmoud G Salloum, Kholood AS Al-Hammad, May Hamza, Geetha Subramaniam, Yanina Singh, Farooq Chaudhary

**Affiliations:** 1 Department of Restorative Dentistry, Jouf University, Sakakah, SAU; 2 Department of Preventive Dentistry, Jouf University, Sakakah, SAU; 3 Department of Pathology, Northern Border University, Arar, SAU; 4 Department of Conservative Dentistry, Jouf University, Sakakah, SAU; 5 Department of Substitutive Dental Sciences, Buraydah Private College, Buraydah, SAU; 6 Department of Dentistry, King Abdulaziz Airbase Hospital, Dhahran, SAU; 7 Department of Prosthetic Dental Sciences, Jouf University, Sakakah, SAU; 8 Department of Health and Life Sciences, INTI International University, Nilai, MYS; 9 Department of Pediatric and Preventive Dentistry, School of Dental Sciences, Sharda University, Greater Noida, IND; 10 Department of Dentistry, Shaheed Zulfiqar Ali Bhutto Medical University, Islamabad, PAK

**Keywords:** student perception, social media, health sciences, distance education, covid-19, blended learning

## Abstract

Background

During the COVID-19 pandemic, universities worldwide pivoted to distance education, primarily online, using various blended learning tools. In the contemporary era, characterized by widespread high-speed internet and the ubiquity of social media (SM), SM has become an essential tool, especially among students. This study aimed to assess the perception, impact, and preferences of various SM platforms for learning among health sciences students in the post-COVID-19 era.

Methodology

The study was conducted at constituent colleges of Jouf University and Northern Border University between January and June 2022. Responses from 560 students were assessed using a self-administered, pre-validated questionnaire comprising 31 questions. These questions addressed students' perceptions, preferences, and learning modes derived from SM. Descriptive and inferential statistics evaluated the influence of SM on student learning.

Results

On average, students spent 3.18 hours daily on SM. YouTube (41.1%) and Instagram (37.1%) emerged as the most preferred platforms for learning. A significant 86.4% of students utilized SM for accessing subject-related texts and watching related videos. Moreover, 78.6% believed that SM platforms enhanced their subject knowledge following lectures. Logistic regression analysis indicated maximum learning benefits for students who used SM between two to three hours daily.

Conclusion

Social media platforms, when used judiciously, can enhance the learning experience for health sciences students in the post-COVID era. While offering opportunities to acquire new knowledge and skills, care must be taken to prevent misuse, abuse, or related health hazards.

## Introduction

For most people who have access to the Internet in many different nations, social media (SM) is quite popular. Universities across the world have started delivering distance education, most of it online, during the COVID-19 pandemic. This is accomplished using a variety of blended learning tools, including synchronous online tutorials. A collection of Web 2.0-inspired, technologically advanced apps that enable the production and exchange of user-generated content is known as SM [1]. In the past several years, SM has seen exponential growth. About 2.65 billion individuals worldwide were reportedly utilizing SM as of 2018. By 2021, there will likely be more than three billion active SM users, or around one-third of the world's population, demonstrating the enormous influence of social connectivity [2]. A cheap, effective, and influential method of facilitating communication online is through the use of SM, via which consumers of SM can browse and share data in a plethora of ways, including by presenting them, exchanging ideas, posting comments, and liking, creating, or altering the web content. It is available in modes like YouTube, Wikipedia, Instagram, Twitter, Facebook, and several others [3,4].

SM use among American adults between 18 to 29 years old surged from 12% in 2005 to 90% in tandem with the broad use of smartphone technology. According to a survey done in 15 countries, higher education students are to blame for the rising use of mobile devices like tablets and smartphones [5]. According to Alanzi and Al-Habib [6], relevant SM platforms utilized by the participants for learning purposes are listed from highest to lowest order according to their usage: YouTube, Twitter, LinkedIn, Snapchat, Instagram, and Facebook. Most of them (58.87%) used these platforms for longer than three hours each day. The majority of those polled (82%), acknowledged that SM may be used to spread information about healthcare quality issues, with YouTube being the channel of choice for this purpose. The use of SM by medical students has also increased significantly. It is giving medical educators new teaching and learning paradigms to consider [7]. A study by Rajeh et al. [8], included 1034 dental students, where the most popular SM network was WhatsApp (97.5%), which was followed by Twitter (85.2%), Snapchat (90.5%), and Instagram (83.4%). SM was utilized for community conversation (55.8%), general information search (63.3%), general idea exchange (63.1%), and amusement (81.4%). According to the majority of publications, social media has a great capacity for boosting contact between learners and their educators [9-12]. It enables customizing teaching to suit each student's learning capabilities and fosters problem-solving in the classroom [13]. It actively addresses instructional issues and helps learners become more involved in the lesson being taught [14]. It also makes possible timely communication, which boosts knowledge accessibility and improves responses during student-teacher interactions [15]. Additionally, resources and relevant content can quickly and effectively be shared through social media in the given time frame. As a result, this form of communication acts as a repository of educational resources, which all students can take advantage of [15].

It is undeniable that SM sites are evolving into a substantial source of scientific data, including news, technical debates, and educational resources. Consumers utilize SM to get health-related consumer evaluations, support a healthy initiative, share their health experience, and establish a health platform or community, according to a poll of 1060 United States (US) adults conducted by a health research institute [16]. Students studying medicine and dentistry can utilize SM to broaden their knowledge, engage in learner-teacher interaction, and participate in the teaching curriculum outside of the classroom. Due to the ease of using the internet instead of actual travel, as well as the ability to engage virtually with a huge number of people through the creation of group discussion forums, users chose traditional educational methods [17].

Social media has nearly permeated the entire world as an effective means of communication. Students at universities utilize social media as their main form of communication. A student's life revolves around studying, tasks, assignments, and projects. However, in today's age of technology, a student's life is additionally impacted by a plethora of social media platforms like Instagram, Facebook, Twitter, and many more, which makes it challenging for students to concentrate on their studies [18,19].

The pandemic has fostered the use of multiple SM platforms in the academic sector, but it has also resulted in the decline of non-verbal communication, the failure of substitute clinical activities, and contacts between educators, learners, and patients. During the COVID-19 epidemic, SM use to further educational objectives had both beneficial and detrimental outcomes [19]. It might be quite helpful to be aware of the benefits and drawbacks of social media in the context of specialized and medical education. A thorough study on using social media for education in the post-COVID era is, however, lacking. The findings of our study could help shed light on continued advantages and existing barriers in relation to the use of SM.

Therefore, this study aims to understand how university students utilize social media in the post-COVID era. The study aims to assess the usage, perception, and preferences associated with SM to enhance learning among the university students in the post-COVID-19 era.

## Materials and methods

The College of Medicine, College of Dentistry, College of Pharmacy, and College of Applied Sciences, Jouf University, and the College of Medicine, College of Pharmacy, and College of Applied Sciences, Northern Border University, Arar, conducted this cross-sectional, non-interventional, survey-based study between January 2022 and March 2022.

After calculating the educational usage of SM, the sample size was approximated using the World Health Organization sample size calculation formula. The sample size formula used for n=Z2P(1-P)d2, where n is the sample size, Z is the statistic corresponding to the level of confidence, P is expected prevalence (it was calculated according to the pilot study), and d is precision (corresponding to effect size). Finally, data were gathered from the estimated sample size of 500. A non-probability convenience sampling technique was utilized to recruit participants in the study. Informed consent was requested from all enrolled participants prior to initiating the study.

The survey questionnaire was inspired by a study on the usage of social media by physicians which identified the factors affecting the sharing of information which helped in lifelong learning and professional development [20]. A modified version of the questionnaire with 31 multiple-choice questions was used for this investigation (Appendix A). A professional team of authors with expertise in creating Google survey forms for cross-sectional studies created this survey on Google. The authors are experts in medical education. It was pre-tested on a chosen sample of students to increase the Google survey form's validity and reliability, which also helped to improve its design. Each class's leader sent a link to the online Google survey form in the WhatsApp group for that particular class. Students, both male and female, were recruited freely to participate in this Google Form survey. The questionnaire included five sections and was completely private and anonymous. Six questions on demographic information, such as gender, academic year, score, college, etc., were included in the first portion. The second section focused on SM usage patterns and included six new questions concerning the frequency of use, kinds of media most and least liked, and use of SM for learning and social interaction. Five questions covering five different perspectives on usage were included in the third section, which asked about perceptions of using SM technologies. The fourth portion, which includes four questions regarding time wasting, different restrictions, etc., talks about SM usage limitations. The final portion discusses how SM is seen in relation to teaching and learning. It comprises 10 questions regarding how people see similar things.

Independent variables

The independent variables were gender, academic year and gross point average (GPA) (GPA < 7/10 and GPA at least 7/10).

Outcome variables

Dependent variables included time spent on SM, and preferred type of SM. The gathered information was transferred to the Statistical Package for Social Sciences (SPSS) version 25 (IBM Corp., Armonk, NY, USA). The descriptive data were used to describe the participant's demographics, use of SM, and attitudes on academic success, career learning, and talent. SPSS version 25 statistical package was employed to analyse categorical data. P<0.05 was considered statistically significant. A logistic regression analysis test was used to assess the regression coefficient of the independent variable on the dependent variable.

## Results

There were a total of 500 participants, out of which 242 were male (48.4%), whereas 258 were female (51.6%). Table 1 demonstrates the students' daily average time spent on SM. Nearly 45.3% of the students used SM for longer than four hours a day, and 3.18 hours per day were calculated as the average amount of time spent.

**Table 1:** Average time spent on social media (SM) per day

Time spent	N	Percentage (%)
1 hour or less	24	4.8
Between 2 and 3 hours	89	17.8
Between 3 and 4 hours	160	31.9
4 hours or more	227	45.3
Average time spent on SM	3.18 hours	

Table 2 shows that the majority of students utilized YouTube (41.1%) and Instagram (37.1%) for educational purposes. Facebook and WhatsApp were the other platforms utilized, accounting for 13.4% and 8.4%, respectively.

**Table 2:** Preferred social media (SM) used for educational purposes

Type of SM	N	Percentage (%)
Facebook	41	8.4
WhatsApp	66	13.4
YouTube	202	41.1
Instagram	182	37.1

Table 3 shows that in the gender-wise analysis, the majority of female participants (52.1%) used SM more when compared to males (47.9%).

**Table 3:** Gender-wise distribution of social media (SM) usage

Gender-wise SM usage	N	Percentage (%)
Females	256	52.1
Males	235	47.9

Table 4 demonstrates that when the academic year-wise distribution was analyzed, the majority usage of SM was seen in the following sequential order: 2nd-year students (37.5), 1st year (23%), 3rd year (16.5%), 4th year (14.9%), and 5th year (8.1%).

**Table 4:** Academic year-wise distribution of social media (SM) usage

Academic year	N	Percentage (%)
Year 1	113	23.0
Year 2	184	37.5
Year 3	81	16.5
Year 4	73	14.9
Year 5	40	8.1

Table 5 demonstrates that 86.4% of the participants were students who used SM platforms to read texts relevant to their fields - in this case, health sciences - as well as to view videos. About 51.4% of the students utilized SM to discover reading material associated with their course themes. A quarter of the students (26.6%) shared videos and links to the instructional resources for their fellow classmates to view. About 39.4% of the participants reported using SM platforms for communication around health literacy. This includes enquiring about and imparting their knowledge of healthcare disciplines. About 14.6% of students utilized this forum to remark and ask questions about various issues in the health sciences.

**Table 5:** Percentage of students using social media (SM) for different educational purposes

Activities of students using SM usage	Percentage (%)
Communication for health education	39.4
Reading text or watching videos	86.4
Finding study materials	51.4
Post videos and links in order to share experience	26.6
Comment/raise Question/answer	14.6

Table 6 depicts that "improving their knowledge following classes" was the main benefit students felt from utilizing SM (78.6%). The second important factor, which 54.6% of the students believed was beneficial to them, was "increasing understanding following practicals or clinics." The students believed that their understanding of "disease prevention" had improved (43.8%), and they believed that SM had taught them about illness management (41.6%).

**Table 6:** Using social media (SM) in different ways to enhance knowledge

Usage of SM for enhancing knowledge	N	Percentage (%)
Improving knowledge after lectures	393	78.6
Improving knowledge after practicals/clinics	273	54.6
Information related to disease prevention	219	43.8
Information related to disease treatment	208	41.6

Table 7 represents the perception of students using SM for teaching purposes, where the result shows that the median value and interquartile range (IQR) were highest among students who believe that class should have a Facebook group, whereas for other purposes like WhatsApp groups, for queries, and for interactive discussion, it was the same.

**Table 7:** Perception of students of applying social media (SM) for teaching purposes

SM for teaching purpose	Median	IQR
Class should have its own Facebook group	3	[3;4]
Class should have its own WhatsApp group	2	[2;3]
SM should be effectively used for queries	2	[2;3]
Interactive discussions should be carried out on SM	2	[2;3]

Table 8 demonstrates the perception regarding SM tools in increasing student interaction for learning, where the majority of students agreed (43.6%) that it helps in increasing the interaction among the students, whereas 28.1% of students gave neutral responses. Only 1.6% strongly disagree with the perception of interaction among students via SM.

**Table 8:** Social media (SM) tools increase student interaction for learning

SM tools increase student interaction for learning	N	Percentage (%)
Strongly disagree	8	1.6
Disagree	38	7.7
Neutral	138	28.1
Agree	214	43.6
Strongly agree	93	18.9

Table 9 demonstrates the pupils' reluctance to utilize SM. When asked if using SM was a waste of time, the majority of students, 34.3%, agreed, while 18.4% strongly agreed. When presented with this question, 32.5% of the class gave an indifferent response. This indicated that the majority considered the excessive use of SM for learning to be a time-waster. When asked if they had trouble using SM for educational reasons because of internet connectivity, the majority of students, 38.9%, answered in the negative. Internet availability was a barrier to learning through SM, according to 26.7% of respondents, with 10% strongly agreeing. The majority of students, 48.7%, agreed that the SM alerts bother them and divert their attention. In contrast to 13% who strongly agreed, 32.7% were ambivalent on the matter. When asked whether using SM for learning causes mental fatigue, 42.3% nodded in agreement, while 13% strongly agreed. When this question was posed, 32.7% of the students responded in the second majority.

**Table 9:** Reasons for reluctance towards using social media (SM)

	SM is wastage of time		Internet availability is a constraint		Notifications are disturbing		SM usage is mentally tiring	
	N	%	N	%	N	%	N	%
Strongly agree	92	18.4	50	10.0	95	19.0	65	13.0
Agree	172	34.3	134	26.7	244	48.7	212	42.3
Neutral	163	32.5	195	38.9	124	24.8	164	32.7
Disagree	52	10.4	88	17.6	28	5.6	46	9.2
Strongly disagree	16	3.2	32	6.4	3	.6	8	1.6
Total	495	98.8	499	99.6	494	98.6	495	98.8

Table 10 shows the analysis regarding the parameter “SM tools increase students' interaction for learning” in relation to the duration of SM usage, gender, academic year, and type of SM platform used. A statistically significant association (B=0.576, p<0.05) was found between the students who used different SM platforms for two to three hours and the learning through SM tools. This group perceived that SM tools increased their interaction for learning by more than four times compared to the students whose usage was less than one hour. Similarly, the students whose usage was between one to two hours and the ones whose usage was more than three hours also perceived that these platforms increased their learning through interaction, and this perception was 1.45 times higher than that of those whose usage was less than one hour.

**Table 10:** Ordinal logistic regression analysis for predicting the variables influenced by the parameter The superscript value describes the constant value which is set to 0 as it is redundant. CGPA: Cumulative gross point average

Parameter	Variables	B	Std. Error	Wald	df	Sig.	95% Confidence Interval	
							Lower Bound	Upper Bound
SM tools increase students' interaction for learning	CGPA	0.124	0.157	0.629	1	0.428	-0.183	0.432
	Duration of SM (1 to 2 hours)	-0.407	0.332	1.496	1	0.221	-1.058	0.245
	Duration of SM (2 to 3 hours)	0.576	0.274	4.433	1	0.035	0.04	1.112
	Duration of SM (More than 3 hours)	-0.232	0.192	1.459	1	0.227	-0.609	0.145
	Duration of SM (Less than 1 hour)	0^a^	.	.	0	.	.	.
	Gender (Male)	-0.226	0.177	1.623	1	0.203	-0.573	0.122
	Gender (Female)	0^a^	.	.	0	.	.	.
	Academic Year (1)	1.057	0.348	9.256	1	0.002	0.376	1.739
	Academic Year (2)	0.87	0.328	7.034	1	0.008	0.227	1.514
	Academic Year (3)	0.51	0.358	2.033	1	0.154	-0.191	1.212
	Academic Year (4)	0.58	0.37	2.451	1	0.117	-0.146	1.306
	Academic Year (5)	0^a^	.	.	0	.	.	.
	Type of SM (Facebook)	-0.403	0.314	1.648	1	0.199	-1.02	0.213
	Type of SM (WhatsApp)	0.366	0.266	1.889	1	0.169	-0.156	0.888
	Type of SM (YouTube)	-0.269	0.188	2.044	1	0.153	-0.638	0.1
	Type of SM (Instagram)	0^a^	.	.	0	.	.	.

The results in the above table also show that the male students felt that these platforms helped their learning more compared to the female students. The results also suggested that there was a statistically significant (B=1.05, p<0.05) relationship between the first- and second-year health sciences students and their learning through SM. This group of students felt that these platforms increased their interaction for learning by nine and seven times, respectively, compared to the fifth-year students. The students felt that YouTube (B=.36, p<0.05) provided twice as much learning compared to Instagram, whereas WhatsApp and Instagram were also more useful platforms compared to Facebook.

## Discussion

The average time that students are spending on SM

The data shows that the students who used different SM platforms for two to three hours perceived that SM tools increased their interaction for learning more than four times compared to the students whose usage was less than one hour. Similarly, the students whose usage was between one to two hours and the ones whose usage was more than three hours also perceived that these platforms increased their learning through interaction and this perception was 1.45 times higher than those whose usage was less than one hour. Hence, we can assume that the SM usage between two to three hours made the students feel that their learning increased with social interaction, through the various SM platforms. In a study by Saadeh et al., the average time spent on SM is three to five hours by the majority of medical students [2]. Another study by Alnjadat et al. [5], reported that the average time spent on SM usage was reported as two to three hours per day. The results of our study showed that the average time spent on SM by students was around 3.18 hours daily. In a study by Deshpande et al. [21], nearly 37% of students reported using the internet for more than four hours each day, and 56% said they use SM more than six times every day. In another study by Kolhar et al., the majority of the students used SM daily for a total of six hours [18]. Bhandarkar et al. [22] reported in their study that the average time spent by 41% of students on SM is around seven hours, while 61.2% of low performers and 51.3% of high performers spent more than three hours every day on SM.

Preferred SM used for educational purposes

In our study, the majority of students (40.3%) used YouTube for educational purposes. The students felt that YouTube provides twice as much learning as compared to Instagram, whereas WhatsApp and Instagram were also more useful platforms compared to Facebook. Similar results were demonstrated by Alsuraihi et al. [7], who found that 41.5% of students were mostly using YouTube. They concluded that it is simpler to search for certain themes on YouTube, share content with others without creating an account, and locate several examples of a given topic. In addition, YouTube was determined to be superior to textbooks and E-Medicine articles in terms of substance, information integration, and user involvement. In the results of Bhandarkar et al. [22], it was seen that the most popular SM platforms were WhatsApp (98.25%) and YouTube (91.75%). According to a systematic review by Guckian et al. [23], undergraduate medical students frequently turn to SM. Although YouTube and WhatsApp were the most popular platforms for instructional information, Facebook was found to be the most widely utilized platform in this category. Between one-third and 50% of pupils regularly used SM for learning. Another study concluded that Facebook was most widely used by medical students and residents, while LinkedIn was most widely used by medical professionals [24].

Gender-wise usage of SM by students

In a study by Alnjadat et al. [5] and D’Souza et al. [4], the majority of users of SM were females (61% and 68.8%, respectively). In a study by Alsuraihi et al. [7], the majority of users were females (60.6%) who believed that SM helped them focus on important topics while studying for their exams. Our results also showed a similar pattern, with female students showing more usage of SM tools compared to their male counterparts, but this difference was negligible compared to the previous studies done in Saudi Arabia. We can derive from the results of our study that the students in our study population in Saudi Arabia felt that SM usage was important in terms of a source of information, irrespective of gender.

Percentage of students using SM for different educational purposes

In a study by Rajeh et al. [8], it was found that among dental students, SM was utilized for enjoyment (81.4%), dentistry education (70.8%), gathering general knowledge (63.3%), exchanging general thoughts (63.1%), and general community conversation (55.8%). The main benefits of adopting SM in education were helping students learn more about various topics, promoting learning engagement, providing a greater opportunity to access new resources, enhancing their capacity for creativity and invention, and enhancing their research abilities. In another study by Bhandarkar et al. [22], students revealed that YouTube offers medical students free educational content, expert training and instructional videos, common-theme vodcasts, and PowerPoint presentations from prestigious colleges and organizations, which helps them improve their visual learning, whereas Whatsapp allows users to contact one another, create peer groups, have discussions and get feedback, and exchange material about medical education from any other SM site, making it effectively a mix of every existing social medium. In the present study, the majority of students used SM for reading text or watching videos.

Perception about the usage of SM

In our study, students believed that Facebook groups were of great importance in terms of learning and sharing knowledge. Similar results were found in the study by Apuke and Iyendo [25], where the students thought casual contact, such as polite conversation among friends, frequently turned into intellectual discussion on course issues. Akbari et al. [26] did research comparing the autonomy, competency, and relatedness of face-to-face group language learning versus Facebook group language learning. They discovered that pupils in the Facebook group tended to be more independent, capable, and sociable. Karal et al. [27] studied how Facebook groups aided in students' language development. Students completed their tasks in these groups, including online chats, written pieces, poetry, and vignettes. The findings, which were based on a variety of data sources from instructors and students, showed that students' language proficiency and their degree of collaboration and engagement with both their teacher and their peers improved.

Using SM in different ways to enhance knowledge

In a study by Diep et al. [1], most students used SM for studying mostly for "sharing learning experiences (71.3%)" and "supplementing information after lectures (67.2%)”. They were utilized by a lesser percentage of people (30.7% to 41.9%) to look for material pertaining to illness prevention, treatment, or consultation. In this study, we found that SM helps improve their knowledge following classes, clinical and practical experiences, and knowledge about disease prevention and treatment. In a systematic review by Guckian et al. [23], between one-third and 50% of undergraduate medical students regularly used SM for learning. In another study by Latif et al. [28], smartphone apps were used by medical students to access online textbooks (70%), podcasts (60%), calculators (75%), online lectures (50%), and take notes (45%). The majority of students use smartphones for education (62.7%), communication (81.7%), and enjoyment (82.5%), according to pertinent research.

SM tools increase student interaction for learning

Student learning improves when they are engaged in active learning [29,30]. The study showed that the majority of the students agreed that the use of SM improved their interaction with peers, which can lead to better learning. A study done in the University of Sharjah, UAE [5], also showed that the students felt that SM helped them interact with other students and access information through collaborative learning. Another study done in a medical school in Morocco showed that the students learn better when interacting with peers and teachers outside the traditional class through SM platforms [24]. The SM platforms can be used to engage students formally as part of the teaching and learning strategy. Suggestions can be made to the academic section of the colleges to incorporate the SM tools as part of the learning strategies for the students. However, this can also have drawbacks. The use of Facebook as an educational tool can have privacy issues, despite using closed groups [29-32]. Other SM platforms can also have similar issues. Hence, if incorporated as a formal learning tool in education, strict guidelines should be developed and implemented for the use of these tools.

Reasons for reservations about using SM

In the present study, the major flaw reported regarding the use of SM usage was time wastage, which was agreed upon by around 34.3% of students, and 38.9% responded to internet availability as a constraint. About 48.7% agreed that notifications are disturbing, and 42.3% agreed that SM is mentally tiring. According to Rajeh et al. [8], major disadvantages of using SM include distraction from studying, increased addictive potential, increased time spent, and concerns over no direct contact with the instructors. In a systematic review, various other reasons were highlighted regarding the adverse effects seen with the usage of SM, i.e., the average amount of time spent on SM by medical students per week was at least six hours. They also reported using Facebook in unhealthy ways, such as holding their urine, skipping meals, and logging in at midnight, which led to restless nights, headaches, back and shoulder pain, and eye irritation. Two studies revealed SM may exacerbate social isolation, while one study connected SM use to an increased risk of anxiety and despair [23].

Academic year-wise distribution of SM usage

The results suggested that the first- and second-year health sciences students believed that these platforms increased their interaction for learning by nine and seven times, respectively, compared to the fifth-year students. This shows that early-year students learn more when interacting with others. Compared to this, fifth-year students become more independent learners and rely more on self-directed learning than collaborative learning through SM. Similar results were seen in the study by Lahiry et al. [29], where the majority of SM users were from the academic year (35.37%). In another study by Alsuraihi et al. [7], most of the SM users were from the third academic year, which they included as medical-phase students who used SM for a deeper understanding of a topic and link to basic and clinical science.

Based on the results of our study, we concluded that there is a positive relationship between the use of SM for two to three hours and the students' learning. This finding is similar to a study done in a medical school in Vietnam where students with similar usage of SM platforms performed better when their cumulative gross point averages (CGPAs) were considered [1]. A study done in three dental schools in Saudi Arabia showed a similar pattern where students felt that moderate usage of SM improved their learning [8]. The students felt that their learning increased with social interaction through the various platforms. We believe that more than three hours resulted in a wastage of time and distraction for the students, whereas less than two hours was too little time for any kind of learning to happen. The students who spent more time on SM probably studied less. Hence, we can conclude that there should be a balance between studying on your own and using SM tools. The literature is not conclusive about the effects of SM on student learning. Some studies have found that SM has a positive impact on student learning, while others have found that students have negative perceptions of their learning education [29,33].

Our study also shows that the early-year students learn more when interacting with others through SM platforms compared to the senior-year students. We can say that in the senior year, students become more independent learners and rely more on self-directed learning more than collaborative learning through SM. The students who enter the first year of any health sciences programme take time to develop their independent learning skills. They use collaborative learning with peers as a support for learning. However, once they progress to the senior years they develop self-regulated learning skills and become more independent learners. Based on this, we can recommend that the students in the early years be supported to develop their independent learning skills.

There were some limitations to the study. The participants voluntarily took part in the study. This may have been a reason for bias towards the results showing an inclination towards the use of SM. The time spans that the students mentioned regarding the use of SM may have been incorrect due to the inability to recall them correctly. Getting a precise time seems difficult since SM tools are frequently used during the day, ranging from short to long time spans. There may have been underreporting of the time spent using SM tools during the day. However, since the participants were all healthcare students who have a hectic study schedule, we can assume that they have less time for SM use, and the time estimates may have been accurate. Another limitation of the study was that the survey was done only in the Northern province of Saudi Arabia, so may not be a true insight about students' perceptions and preferences from all other universities situated in different parts of Saudi Arabia.

## Conclusions

This is the first study done in northern Saudi Arabia amongst healthcare students to identify the pattern of SM usage and its association with the students' learning. SM is still considered a mode of learning in the post-COVID-19 era that helps health sciences students learn well if used appropriately. Most of the students in the setting use different SM platforms for learning, with Instagram and YouTube being the most utilized. Students felt that SM usage of two to three hours daily maximized their learning. The findings of our study can be considered by the university administration in order to incorporate the use of SM platforms formally into teaching and learning strategies. We feel that with appropriate rules and regulations, these platforms can be used to enhance student learning in addition to traditional methodologies.

The COVID-19 pandemic has had significant effects on education; as a result, there seems to be an increase in the usage of social media for education during an epidemic. Overall, the reaction from the educators was favorable; the benefits of adopting social media for learning outweighed the drawbacks. Although more research is required, these results imply that social media's special qualities and accessibility can be quite helpful in educational activities. Therefore, educational institutions can use hybrid blended learning, which combines in-person and online instruction, to give more students the advantage of being able to take courses that may not be offered near them and to give them the benefit of learning at flexible times.

## Appendices

APPENDIX A Questionnaire

Section 1 (Demographic information)

1.      E-mail:

2.      Institute:

3.      College:

o   College of Medicine

o   College of Dentistry

o   College of Applied Sciences

o   Others

4.      Gender

o   Male

o   Female

5.      CGPA of the last academic year

-------------------------------------

6.      Academic year: (Mark only one)

o   Year 1

o   Year 2

o   Year 3

o   Year 4

o   Year 5

Section 2 (PATTERN OF SOCIAL MEDIA USAGE)

1.      Duration of social media usage (Mark only one)

o   Less than one hour

o   Between 1 and 2 hours

o   Between 2 and 3 hours

o   3 or more hours

2.      Type of social media usage: (check all that apply)

o   Facebook

o   YouTube

o   WhatsApp

o   Instagram

3.      Most preferred social media platform: (Mark only one)

o   Facebook

o   YouTube

o   WhatsApp

o   Instagram

4.      Least preferred social media platform: (Mark only one)

o   Facebook

o   YouTube

o   WhatsApp

o   Instagram

5.      Usage of social media for activities: (Check all that apply)

o   For reading and watching videos

o   Finding study materials

o   Comments/raising questions

o   Posts to share experiences

o   Communication for learning

6.      Use of social media for learning: (Check all that apply)

o   Improving knowledge after lectures

o   Improving knowledge after practical/clinical

o   Information related to disease prevention

o   Information related to disease treatment

SECTION 3 (PERCEPTION REGARDING USE OF SOCIAL MEDIA TOOLS)

7.      To share knowledge with others (Mark only one)

o   Strongly agree

o   Agree

o   Neutral

o   Disagree

o   Strongly disagree

8.      Downloading and uploading files on social media is convenient (Mark one only)

o   Strongly agree

o   Agree

o   Neutral

o   Disagree

o   Strongly disagree

9.      Useful to learn from others through social media platforms (Mark one only)

o   Strongly agree

o   Agree

o   Neutral

o   Disagree

o   Strongly disagree

10.  Useful as an online discussion tool (Mark one only)

o   Strongly agree

o   Agree

o   Neutral

o   Disagree

o   Strongly disagree

11.  Prefer to work with groups on social media (Mark one only)

o   Strongly agree

o   Agree

o   Neutral

o   Disagree

o   Strongly disagree

SECTION 4 (LIMITATIONS OF THE USAGE OF SOCIAL MEDIA)

12.  The usage of social media platforms results in wastage of time: (Mark one only)

o   Strongly agree

o   Agree

o   Neutral

o   Disagree

o   Strongly disagree

13.  Internet availability is a possible constraint in using social media platforms for learning: (Mark one only)

o   Strongly agree

o   Agree

o   Neutral

o   Disagree

o   Strongly disagree

14.  Social media notifications are a potential source of bother: (Mark one only)

o   Strongly agree

o   Agree

o   Neutral

o   Disagree

o   Strongly disagree

15.  Usage of social media platforms is mentally tiring: (Mark one only)

o   Strongly agree

o   Agree

o   Neutral

o   Disagree

o   Strongly disagree

SECTION 5 (PERCEPTION ABOUT LEARNING THROUGH SOCIAL MEDIA PLATFORMS)

16.  Class should have its own Facebook page: (Mark one only)

o   Strongly agree

o   Agree

o   Neutral

o   Disagree

o   Strongly disagree

17.  Class should have its own WhatsApp group: (Mark one only)

o   Strongly agree

o   Agree

o   Neutral

o   Disagree

o   Strongly disagree

18.  Students can use social media effectively for interacting and asking questions: (Mark one only)

o   Strongly agree

o   Agree

o   Neutral

o   Disagree

o   Strongly disagree

19.  Interactive discussions can be done through social media (groups, pages, blogs, etc.) (Mark one only)

o   Strongly agree

o   Agree

o   Neutral

o   Disagree

o   Strongly disagree

20.  Social media network meets students’ needs and interests: (Mark one only)

o   Strongly agree

o   Agree

o   Neutral

o   Disagree

o   Strongly disagree

21.  Social networking is an effective way for the students to connect: (Mark one only)

o   Strongly agree

o   Agree

o   Neutral

o   Disagree

o   Strongly disagree

22.  I enjoy my time when using social media platforms: (Mark one only)

o   Strongly agree

o   Agree

o   Neutral

o   Disagree

o   Strongly disagree

23.  Social media tools increase student’s interaction for learning: (Mark one only)

o   Strongly agree

o   Agree

o   Neutral

o   Disagree

o   Strongly disagree

24.  Social media enables me to share my knowledge: (Mark one only)

o   Strongly agree

o   Agree

o   Neutral

o   Disagree

o   Strongly disagree

25.  By using social media platforms, students can personalize their learning needs: (Mark one only)

o   Strongly agree

o   Agree

o   Neutral

o   Disagree

o   Strongly disagree
